# Prophylactic anticoagulation to prevent venous thromboembolism in traumatic intracranial hemorrhage: a decision analysis

**DOI:** 10.1186/cc8980

**Published:** 2010-04-20

**Authors:** Damon C Scales, Jay Riva-Cambrin, Dave Wells, Valerie Athaide, John T Granton, Allan S Detsky

**Affiliations:** 1 Department of Critical Care Medicine, Sunnybrook Health Sciences Centre, 2075 Bayview Avenue, Room D108, Toronto, ON M4N 3M5, Canada; 2 Institute for Clinical Evaluative Sciences, 2075 Bayview Avenue, Room G157, Toronto, ON M4N 3M5, Canada; 3 Interdepartmental Division of Critical Care Medicine and Department of Medicine, University of Toronto, 30 Bond Street, Toronto, ON M5B 1W8, Canada; 4 Department of Neurosurgery, Primary Children's Medical Centre, 100 N. Mario Capecchi Dr., Suite. 1475, Salt Lake City, Utah 84113, USA; 5 Department of Neurosurgery, University of Utah, 30 N. 1900 E, Salt Lake City, Utah 84132, USA; 6 Faculty of Medicine, University of British Columbia, 17 - 2194 Health Sciences Mall, Vancouver, BC V6T 1Z3, Canada; 7 Departments of Medicine and Critical Care, University Health Network, 11-1170 CSB, 585 University Ave, Toronto, ON M5G 2C4, Canada; 8 Department of Medicine, Mount Sinai Hospital, 600 University Avenue, Suite 429, Toronto, ON M5G 1X5, Canada; 9 Department of Health Policy, Management and Evaluation, University of Toronto, 155 College Street, Suite 425, Toronto, ON M5T 3M6, Canada

## Abstract

**Introduction:**

Patients with intracranial hemorrhage due to traumatic brain injury are at high risk of developing venous thromboembolism including deep vein thrombosis (DVT) and pulmonary embolism (PE). Thus, there is a trade-off between the risks of progression of intracranial hemorrhage (ICH) versus reduction of DVT/PE with the use of prophylactic anticoagulation. Using decision analysis modeling techniques, we developed a model for examining this trade-off for trauma patients with documented ICH.

**Methods:**

The decision node involved the choice to administer or to withhold low molecular weight heparin (LMWH) anticoagulation prophylaxis at 24 hours. Advantages of withholding therapy were decreased risk of ICH progression (death, disabling neurologic deficit, non-disabling neurologic deficit), and decreased risk of systemic bleeding complications (death, massive bleed). The associated disadvantage was greater risk of developing DVT/PE or death. Probabilities for each outcome were derived from natural history studies and randomized controlled trials when available. Utilities were obtained from accepted databases and previous studies.

**Results:**

The expected value associated with withholding anticoagulation prophylaxis was similar (0.90) to that associated with the LMWH strategy (0.89). Only two threshold values were encountered in one-way sensitivity analyses. If the effectiveness of LMWH at preventing DVT exceeded 80% (range from literature 33% to 82%) our model favoured this therapy. Similarly, our model favoured use of LMWH if this therapy increased the risk of ICH progression by no more than 5% above the baseline risk.

**Conclusions:**

Our model showed no clear advantage to providing or withholding anticoagulant prophylaxis for DVT/PE prevention at 24 hours after traumatic brain injury associated with ICH. Therefore randomized controlled trials are justifiable and needed to guide clinicians.

## Introduction

It is estimated that more than 1.5 million people in the United States sustain traumatic head injury each year [1]. Radiologic evidence of intracranial hemorrhage at the time of presentation is present in up to 45% of cases and is associated with a markedly poorer prognosis [2,3]. Traumatic intracranial hemorrhage encompasses cerebral contusion, subdural hematoma, subarachnoid hemorrhage, epidural hematoma and intracerebral hemorrhage. These are characterized by a relatively high risk of bleeding progression, especially within the first 24 hours [2,4-6].

Traumatic intracranial hemorrhage is also associated with a high risk of thromboembolic complications [7]. This risk is related to the immobility of head-injured patients arising from the underlying neurologic lesion itself, concomitant injuries following trauma, or the use of sedatives and neuromuscular blocking agents. The reported incidence of deep vein thrombosis (DVT) ranges between 18 and 58% in the absence of anticoagulant prophylaxis [8-11]. DVT is associated with increased morbidity and mortality, including risk of fatal pulmonary embolism (PE) [12].

Anticoagulant prophylaxis to prevent DVT is recommended for trauma patients without intracranial or other serious hemorrhage [13]. This therapy is also effective in post-operative neurosurgical patients undergoing brain tumour excision [12]. Only one small quasi-randomized clinical trial has studied anticoagulant prophylaxis versus pneumatic compression devices in patients with intracranial hemorrhage following trauma. This trial found no benefit or harm associated with use of anticoagulant prophylaxis, but only had sufficient sample size to detect very large differences in rates of venous thromboembolism or intracranial hemorrhagic progression [14]. One observational study reported rates of hemorrhagic progression in 150 patients with intracranial hemorrhage that were treated with low molecular weight heparin prophylaxis 24 hours after the initial head injury [15]. One quarter already had radiological evidence of intracranial hemorrhagic progression at the time anticoagulant prophylaxis was started, but only six patients (4%) subsequently developed progression in the 24 hours following initiation of anticoagulation prophylaxis. The study lacked a control group limiting inferences regarding the safety and efficacy of this therapy.

Many physicians report routinely withholding anticoagulation prophylaxis in patients with traumatic intracranial hemorrhage because of concerns that it may increase the risk of potentially devastating intracranial hemorrhagic progression [16,17]. Observational studies have also documented variable practice patterns for anticoagulation prophylaxis in such patients. A small retrospective study of 88 patients with traumatic brain injury observed that only 42% ever received low-molecular weight heparin prophylaxis, and the mean time to initiation of therapy was 14 days [18]. Similarly, a multicentre retrospective study of trauma patients with ICU length of stay greater than seven days found that prophylaxis was initiated within two days in only 25% of patients, and another quarter did not receive prophylaxis until at least one week following injury [19]. Thus, the use and timing of anticoagulation prophylaxis in these patients remains uncertain and controversial, and most are treated with more conservative and less effective measures such as graduated compression stockings, intermittent pneumatic compression devices, and physiotherapy [13,16].

The lack of persuasive evidence to guide decisions about using anticoagulant prophylaxis in patients with traumatic intracranial hemorrhage implies that clinicians must make decisions based on their own assessments of the risks and benefits. Our objective was to explore the decision to use or withhold anticoagulant prophylaxis in a common clinical scenario characterized by competing risks of hemorrhagic versus thromboembolic complications. We used decision analysis modeling techniques to compare the risks of progression of intracranial hemorrhage following trauma versus the potential benefits of reducing venous thromboembolism with anticoagulant prophylaxis. Decision analysis involves identifying the most important available clinical choices and determining probabilities of all important potential outcomes following each of these choices [20]. This methodology has been used previously to evaluate the risks and benefits of anticoagulant prophylaxis in neurosurgical patients undergoing craniotomy [21], but to our knowledge has not specifically been used to evaluate patients with traumatic intracranial hemorrhage.

## Materials and methods

### Probabilities and utilities

We searched Medline (OVID Technologies, New York, NY, USA; 1950 to March 2008) for studies concerning intracranial hemorrhage and anticoagulant thromboprophylaxis using the search terms *brain injury, acute; craniocerebral trauma; cerebral hemorrhage, traumatic*, *anticoagulation*, and *heparin*. We used natural history studies and controlled clinical trials performed in both post-operative neurosurgical and trauma patients to estimate the incidence of DVT [9-12,22]. We estimated the risk of hemorrhagic progression using natural history studies that reported the absolute risk at 24 hours following the neurological injury [15]. The risk of systemic bleeding complications was estimated from rates of major gastrointestinal hemorrhage in critically-ill patients [23]. We used clinical trials of anticoagulation prophylaxis in elective neurosurgical patients to estimate the effectiveness of anticoagulant prophylaxis in preventing DVT and its potential impact on subsequent intracranial bleeding [12,24,25].

Table 1 summarizes the probabilities used in the decision analysis. We obtained utility values (Table 2) from previous publications and the Cost-Effectiveness Analysis Registry from the Institute for Clinical Research and Health Policy Studies, Tufts Medical Center [26-35]. We considered the baseline utility for our study (0.96) to be less than a perfect state of health (1.00) because all patients in our model would have already suffered a major trauma with associated head injury [36]. We subtracted utilities associated with temporary states in our model, such as deep vein thrombosis, from the baseline utility, but we considered events having long-term morbidity, such as disabling neurological deficit, by multiplying the associated utility by the baseline utility [28]. The most severe event that could be encountered in the model was death (utility = 0), and the worst outcome that could be experienced while alive was a severe disabling neurological deficit after progression of intracranial hemorrhage.

**Table 1:** Probabilities used in the decision analysis

Variables	Baseline probability	Range of plausible probabilities	Threshold value within published range	Threshold from 0 to 1
Probability of DVT	0.32 [12]	0.18 to 0.50 [[9] to [11,12]]	No	Yes (> 0.6*)
Probability of PE following development of DVT	0.1[42,43]	0.01 to 0.18 [22,24,44,45]	No	Yes (> 0.3*)
Probability of death from PE	0.17 [46]	0 to 0.50 [47]	No	No
Probability of a CNS bleed on LMWH	0.108 [15]	0.03 to 0.23 [24,44]	No	Yes (> 0.017^+^)
Effectiveness of LMWH in preventing DVT (e)	0.47 [12]	0.33 to 0.82 [12,42]	Yes (> 0.8*)	Yes (> 0.8*)
Probability of death from a CNS bleed	0.105 [48]	0.08 to 0.3 [49,50]	No	No
Probability of disabling neurological deficit after CNS bleed	0.17 [51]	0.16 to 0.33 [52,53]	No	No
Probability of an ICU-related systemic bleed	0.035 [23]	0.027 to 0.046 [23]	No	No
Probability of death from ICU-related systemic bleed	0.02 [23]	0.001 to 0.035 [23,46]	No	No
Effectiveness of not receiving LMWH in reducing CNS bleeds	0.315 [4,15]	0.001-0.99 [4-6,23,47,54]	Yes (> 0.05^+^)	Yes (> 0.05^+^)
Effectiveness of not receiving LMWH in reducing ICU-related systemic bleeds	0.146	0 to 0.66 [8,12,55]	No	No

Table of probabilities and plausible ranges used for the decision analysis. The last two columns indicate variables for which a threshold value was identified in one-way sensitivity analysis. Values with an asterisk (*) indicate a threshold value above which providing anticoagulant prophylaxis becomes the preferred strategy. Values with a plus (+) indicate a threshold value above which withholding anticoagulant prophylaxis becomes the preferred strategy.

CNS: central nervous system; DVT: deep vein thrombosis; ICU: intensive care unit; PE: pulmonary embolism

**Table 2:** Utilities used in the decision analysis

Utility	Baseline value	Published ranges	Threshold value within published range	Threshold value reached between 0 and 1
Baseline	0.96 [36]	0.95 to 1.0*	No	No
LMWH prophylaxis	0.99 [27]	0.9 to 1.0*	No	No
Living with DVT	0.95 [27]	0.92 to 1.0*	No	Yes (> 0.9^+^)
Living with PE	0.7 [35]	0.6 to 0.9 [32]	No	No
Living with massive systemic bleed	0.7 [27]	0.6 to 0.8*	No	No
Living with a non-disabling ICH	0.75 [33,34]	0.6 to 0.8 [33,34,56,57]	No	No
Living with a disabling ICH	0.39 [33,34]	0.14 to 0.43 [33,34,57]	No	No

Table of utilities and plausible ranges used for the decision analysis. Ranges with an asterisk (*) were generated from a consensus of authors. The last two columns indicate variables for which a threshold value was identified in one-way sensitivity analysis. Values with a plus (+) indicate a threshold value above which withholding anticoagulant prophylaxis becomes the preferred strategy. DVT: deep vein thrombosis; ICH: intracranial hemorrhage; LMWH: low molecular weight heparin; PE: pulmonary embolism

We also performed sensitivity analyses considering estimates of benefits and risks of anticoagulant prophylaxis reported on our recent survey of Canadian neurosurgeons and neuro-intensivists [17]. We chose the three most frequent estimates of risk-category selected by survey respondents for risk of intracranial hemorrhagic progression and for risk of DVT/PE with and without anticoagulant prophylaxis. We considered the upper and lower boundaries defined by these three response categories to be the range of plausible values, and chose the median from the most frequent response risk-category to be the point estimate for sensitivity analyses.

### Decision node

The base case for our model was an adult trauma patient (≥ 18 years of age) with intracranial hemorrhage. The single decision node was whether or not to treat with LMWH anticoagulant prophylaxis at 24 hours after head injury and continued until hospital discharge. We assumed that all patients were treated with graduated compression stockings, but we did not consider sequential compression devices because our recent survey of Canadian practice showed these are seldom used in this patient population [17].

We considered the main advantage of anticoagulation prophylaxis to be a reduction in the risk of major thromboembolic complications, including DVT, pulmonary embolism, and death. We considered the disadvantages to be an increased risk of intracranial hemorrhagic progression, other bleeding complications, and the pain of subcutaneous injections. A *disabling neurological deficit *was defined as being equivalent to a stroke with a Modified Rankin Score ≥ 3, and a *non-disabling neurological deficit *was defined as being equivalent to a stroke with a Modified Rankin Score ≤ 2 [37]. We considered the risks of developing major outcomes during the first thirty days of hospital admission.

### Tree structure

**Figure 1:**
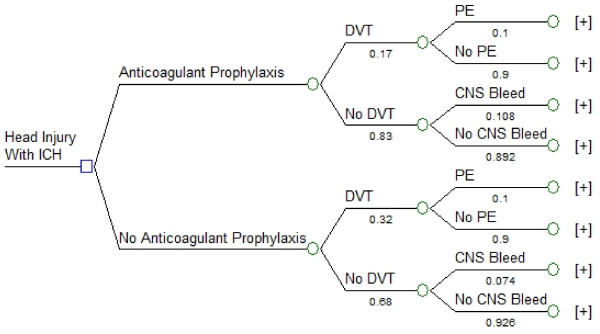
**Decision node and anticoagulation subtree**. Decision analysis tree demonstrating two strategies: providing anticoagulant prophylaxis at 24 hours to an adult patient following head injury with intracranial hemorrhage (ICH), or withholding anticoagulant prophylaxis. A linkage term representing the complement of the effectiveness of anticoagulant prophylaxis for reducing the risk of DVT was used to link the main subtrees. This effectiveness term was calculated as follows: effectiveness of anticoagulant prophylaxis = ((probability of DVT without LMWH) - (probability of DVT with LMWH))/(probability of DVT without LMWH). The square node at the extreme left represents the decision node, the circles represent chance nodes, and the plus signs at the far right indicate the presence of additional branches. Numerical values under each branch are the baseline probabilities used at each chance node. CNS Bleed: progression of intracranial hemorrhage; DVT: deep vein thrombosis; PE: pulmonary embolism.

**Figure 2:**
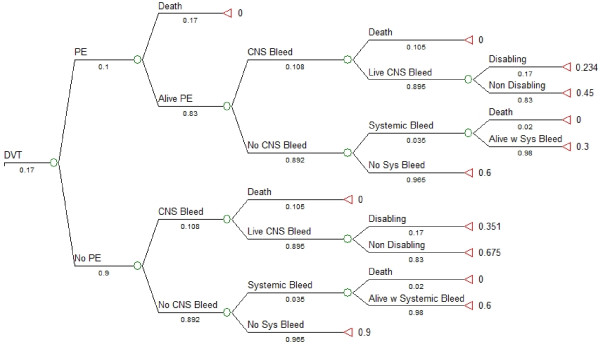
**Deep vein thrombosis subtree with systemic bleeding subtrees**. Deep vein thrombosis (DVT) subtree showing possible outcomes following development of DVT (tree shown is for strategy of anticoagulant prophylaxis). A linkage term representing the complement of the effectiveness of withholding anticoagulant prophylaxis for reducing the risk of systemic bleeding was used to link the distal subtrees. This effectiveness term was calculated as follows: effectiveness of withholding anticoagulant prophylaxis = ((probability of systemic bleeding with LMWH) - (probability of systemic bleeding without LMWH))/(probability of systemic bleeding with LMWH). The circles represent chance nodes, and the triangles on the far right represent the outcome measure, expected utility. Numerical values under each branch are the baseline probabilities used at each chance node for the baseline case with administration of anticoagulant prophylaxis. Values to the right of each triangle are the final expected values (utility) for each state. Alive PE: survival after a pulmonary embolism; Alive w Systemic Bleed: survival after a systemic bleeding complication; CNS Bleed: progression of intracranial hemorrhage; Disabling: disabling neurological deficit; DVT: deep vein thrombosis; Non Disabling: non-disabling neurological deficit; No Sys Bleed: no systemic bleeding complication; PE: pulmonary embolism; Systemic Bleed: hemorrhagic complication not involving central nervous system.

**Figure 3:**
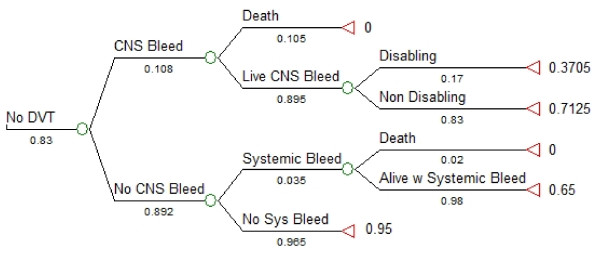
**No deep vein thrombosis (DVT) subtree showing possible outcomes without development of DVT**. The tree shown is for strategy of anticoagulant prophylaxis. A linkage term representing the complement of the effectiveness of withholding anticoagulant prophylaxis for reducing the risk of progression of ICH progression was used to link the main subtrees. This effectiveness term was calculated as follows: effectiveness of withholding anticoagulant prophylaxis = ((probability of ICH progression with LMWH) - (probability of ICH progression without LMWH))/(probability of ICH progression with LMWH). The circles represent chance nodes, and the triangles on the far right represent the outcome measure, expected utility. Numerical values under each branch are the baseline probabilities used at each chance node for the baseline case and with administration of anticoagulant prophylaxis. Values to the right of each triangle are the final expected values (utility) for each state. Alive w Systemic Bleed: survival after a systemic bleeding complication; CNS Bleed: progression of intracranial hemorrhage; Disabling: disabling neurological deficit; DVT: deep vein thrombosis; Live CNS Bleed: survival after progression of intracranial hemorrhage; Non Disabling: non-disabling neurological deficit; No Sys Bleed: no systemic bleeding complication; Systemic Bleed: gastrointestinal bleeding.

We used TreeAge Pro 2008 (TreeAge Software Inc., Williamstown, MA, USA) to perform the decision analysis. The subtrees in our model are shown in Figures 1, 2 and 3. We created a linkage term to represent the effectiveness of anticoagulant prophylaxis at reducing the risk of DVT, calculated as follows: effectiveness of anticoagulant prophylaxis = ((probability of DVT without LMWH) - (probability of DVT with LMWH))/(probability of DVT without LMWH) (Figure 1) [38]. We used the complement of this effectiveness term (1-effectiveness) in the anticoagulant prophylaxis subtree to ensure that no probabilities would ever have values greater than 1.0, and to guarantee that the risk of DVT would always be highest in patients not receiving anticoagulation. Similarly, we used two linkage terms within the *DVT *subtree (Figure 2) and the *No DVT *subtree (Figure 3) to reflect effectiveness of withholding anticoagulant prophylaxis to reduce the risk of systemic bleeding complications or of intracranial bleeding complications. Symmetry was maintained at all major branches of our model and trade-offs at each node were identical for both strategies [38]. The expected utility for each branch was determined by multiplying all of the probabilities along the branches to obtain the probability of being in each state of the terminal nodes. These products of probabilities were then used as the weights to derive the expected value by multiplying the product of probabilities by each of the utilities and then summing these over all outcomes for each branch [39]. Sensitivity analyses were performed across all plausible values for all variables.

## Results

**Figure 4:**
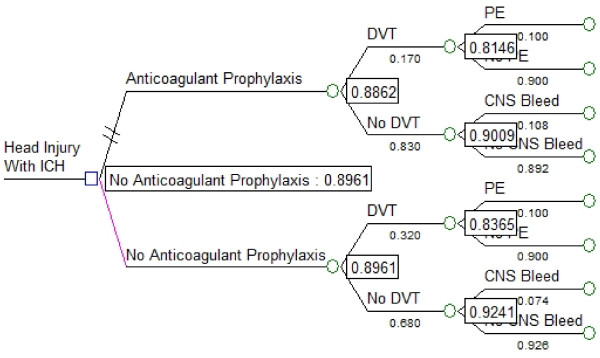
**Results of decision analysis**. The square node at the extreme left represents the decision node and the circles represent chance nodes. Numbers in boxes are the final calculated expected value at each chance node. The overall expected value associated with withholding anticoagulation prophylaxis (0.8961) is similar to that associated with the anticoagulant prophylaxis strategy (0.8862), indicating the choice is a *toss-up*. CNS Bleed: progression of intracranial hemorrhage; DVT: deep vein thrombosis; ICH: intracranial hemorrhage; PE: pulmonary embolism.

**Figure 5:**
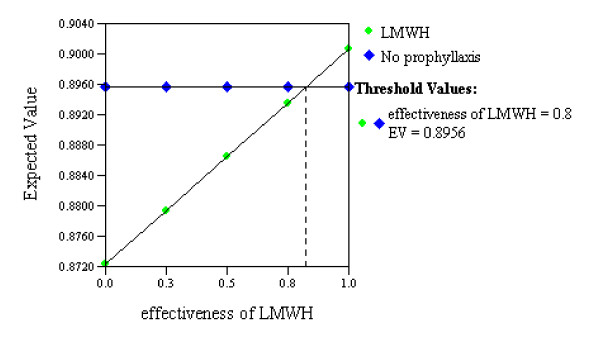
**Sensitivity analysis on effectiveness of low molecular weight heparin at preventing deep venous thrombosis**. One-way sensitivity analysis in which the relative effectiveness of low molecular weight heparin (LMWH) at preventing deep vein thrombosis (DVT) is varied (small green diamonds). The two strategies (LMWH versus no prophylaxis) are seen to have equivalent expected values (utilities) at the point (threshold value) where the two lines intersect. This analysis suggests that providing LMWH at 24 hours after traumatic brain injury with intracranial hemorrhage would become the preferred strategy only if it was more than 80% effective at preventing DVT.

**Figure 6:**
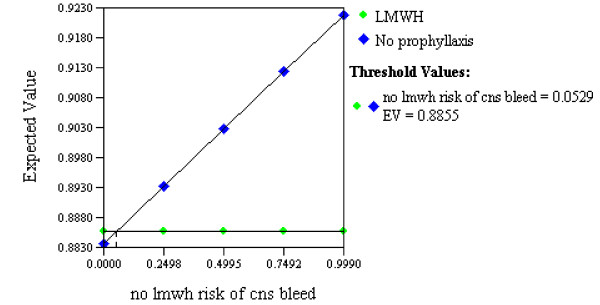
**Sensitivity analysis on effectiveness of no anticoagulant prophylaxis at preventing progression of intracranial hemorrhage**. One-way sensitivity analysis in which the relative effectiveness of *withholding *low molecular weight heparin (LMWH) to prevent progression of intracranial hemorrhage (CNS bleed) is varied (large blue diamonds). The two strategies (LMWH versus no prophylaxis) are seen to have equivalent expected values (utilities) at the point (threshold value) where the two lines intersect. This analysis suggests that LMWH would become the preferred strategy only if it increased the risk of intracranial hemorrhagic progression by no more than 5% above the baseline risk.

The expected value associated with withholding anticoagulation prophylaxis (0.90) was similar to that associated with the LMWH strategy (0.89; Figure 4). A threshold value was reached within our range of estimates for the effectiveness of anticoagulant prophylaxis for preventing DVT (threshold 0.80, range of estimates 0.33 to 0.82; Figure 5), suggesting that providing LMWH would have a greater expected value than withholding LMWH only if its effectiveness for preventing DVT exceeded 80%. Similarly, the variable representing the effectiveness of withholding anticoagulant prophylaxis for reducing the risk of intracranial hemorrhagic progression also reached a threshold value within our range of estimates (threshold 0.05, range of estimates 0.001 to 0.990; Figure 6), suggesting LMWH would become the preferred strategy if it increased the risk of ICH progression by no more than 5% above the baseline risk.

We performed sensitivity analyses using the most frequently reported risk estimates obtained from our survey of Canadian neurosurgeons and neurointensivists. These analyses did not produce any additional threshold variables and the results did not qualitatively change our findings. We also considered sequential compression devices as a third strategy for preventing DVT. For this analysis, we considered the effectiveness of sequential compression devices for preventing DVT to be between that of LMWH and no anticoagulation prophylaxis (effectiveness 0.19, equivalent to point estimate of DVT incidence of 26%) and the risk of intracranial hemorrhagic progression to be the same as no anticoagulation prophylaxis. The expected value of the sequential compression device strategy was 0.90, similar to that of the other strategies (results not shown, but available upon request).

## Discussion

Our results suggest that the decision of whether or not to use anticoagulant thromboprophylaxis 24 hours after traumatic intracranial hemorrhage is a *toss-up*. Although the *no prophylaxis *strategy was associated with a slightly higher expected value (0.90 versus 0.89), this difference is unlikely to be clinically important. For example, the magnitude of this difference is equivalent to the disutility associated with administering a subcutaneous injection of anticoagulant prophylaxis.

Despite the similar expected values associated with each strategy, anticoagulant thromboprophylaxis became the preferred approach only in situations where the incremental risk of hemorrhagic progression was very low, or when its effectiveness in preventing venous thromboembolism was very high. These situations reflect the limits of our plausible risk estimates, and therefore seem unlikely to apply to most clinical situations. Considering the uncertainty, routinely withholding anticoagulant prophylaxis seems an appropriate strategy based on our findings, especially in the early phase when risk of hemorrhagic progression is perceived to be highest [17].

Our results are consistent with the findings of our recent survey of Canadian practice [17]. Of the 160 intensivists and neurosurgeons surveyed, almost two-thirds (60%) of intensivists and neurosurgeons indicated they would use, at some time, anticoagulant thromboprophylaxis in patients with intracranial hemorrhage due to traumatic brain injury. However, only one-third (34%) of these respondents reported that they would start this thromboprophylaxis within two days of the surgery, reflecting their concerns about risk of hemorrhagic progression. However, slightly more than half (57%) would start anticoagulation within four days and most (80%) by one week, suggesting that the perceived risk of hemorrhagic progression decreases over time. We lacked sufficient data to evaluate the risks and benefits of initiating anticoagulant prophylaxis after more than 24 hours, but this could be the topic of future research. We only considered anticoagulant prophylaxis with LMWH rather than unfractionated heparin, reasoning that most available studies in neurosurgical patients used the former and that direct comparisons of these two types of prophylaxis have yielded similar results [40].

Instead of considering anticoagulant prophylaxis, some clinicians may choose to routinely screen patients with ultrasound or other imaging modalities to identify patients with DVT who require treatment with vena cava filters or full-dose anticoagulation, but we did not consider this to be a strategy for DVT prophylaxis. Similarly, depending on available resources some clinicians may choose to use sequential compression devices to prevent DVT while withholding anticoagulation to avoid intracranial hemorrhagic progression. However, this strategy would have important cost considerations and did not yield an apparent benefit in our decision analysis or reduce DVT incidence in a recent large clinical trial in stroke patients [41].

A limitation of all decision analyses is that they rely on having accurate estimates of probabilities of outcomes following clinical choices, and such estimates often are lacking in the literature for the clinical scenario of interest. For example, a limitation of our decision analysis was the difficulty quantifying the incremental risk of intracranial hemorrhagic progression at 24 hours with anticoagulant prophylaxis. Our only estimate was derived from a single prospective study that lacked a control group, so we used a very wide confidence interval in sensitivity analysis. These analyses suggest that anticoagulant prophylaxis would still only become the most effective strategy if the true incremental risk of hemorrhagic progression were less than 5% above baseline. Furthermore, the expected values associated with clinical choices in a decision analysis will depend on the utilities that are assigned to each clinical outcome, and different patients and clinicians may weigh such outcomes differently in the real world.

## Conclusions

Our model showed no clear advantage to providing or withholding anticoagulant prophylaxis for DVT/PE prevention at 24 hours after traumatic brain injury associated with intracranial hemorrhage. In the context of such a *toss-up*, and given that the most disastrous event (other than death) encountered in this model is the development of a disabling neurological deficit, we would recommend against the use of routine anticoagulant prophylaxis at 24 hours for these patients. However, considering that both strategies were associated with nearly equivalent expected values, our model would suggest that it should be ethical to conduct a randomized, controlled, clinical trial to evaluate the safety and efficacy of using anticoagulant prophylaxis in patients with traumatic brain injury and intracranial hemorrhage.

## Key messages

• Patients with traumatic brain injury associated with intracranial hemorrhage are at high risk for developing venous thromboembolism

• Anticoagulation prophylaxis is proven to decrease the risk of venous thromboembolism in other patient groups, but may increase the risk of progression of intracranial hemorrhage

• The decision to provide anticoagulation prophylaxis to these patients therefore represents a trade-off that can be examined using decision analysis techniques

• Our decision analysis model showed no clear advantage to providing or withholding anticoagulant prophylaxis for thromboembolic prophylaxis at 24 hours after traumatic brain injury associated with intracranial hemorrhage

• Randomized controlled trials of this study question are justifiable and needed to guide clinicians

## Abbreviations

CNS: central nervous system; DVT: deep vein thrombosis; ICH: intracranial hemorrhage; ICU: intensive care unit; LMWH: low-molecular weight heparin; PE: pulmonary embolism; SYS: systemic bleeding.

## Competing interests

The authors declare that they have no competing interests.

## Authors' contributions

DCS, JRC, JTG and ASD conceived of and designed the manuscript. DCS, JRC, VA and DW were responsible for data acquisition. DCS, JRC, DW and ASD were responsible for analysis and interpretation. DCS, JRC, DW, VA, JTG and ASD drafted and revised the manuscript. All authors read and approved the final manuscript.
